# Correcting the R165K substitution in the first voltage-sensor of Ca_V_1.1 right-shifts the voltage-dependence of skeletal muscle calcium channel activation

**DOI:** 10.1080/19336950.2019.1568825

**Published:** 2019-01-14

**Authors:** Yousra El Ghaleb, Marta Campiglio, Bernhard E. Flucher

**Affiliations:** Department of Physiology and Medical Physics, Medical University Innsbruck, Innsbruck, Austria

**Keywords:** Voltage-gated calcium channel, Ca_V_1.1, voltage-sensing, skeletal muscle, dysgenic myotubes

## Abstract

The voltage-gated calcium channel Ca_V_1.1a primarily functions as voltage-sensor in skeletal muscle excitation-contraction (EC) coupling. In embryonic muscle the splice variant Ca_V_1.1e, which lacks exon 29, additionally function as a genuine L-type calcium channel. Because previous work in most laboratories used a Ca_V_1.1 expression plasmid containing a single amino acid substitution (R165K) of a critical gating charge in the first voltage-sensing domain (VSD), we corrected this substitution and analyzed its effects on the gating properties of the L-type calcium currents in dysgenic myotubes. Reverting K165 to R right-shifted the voltage-dependence of activation by ~12 mV in both Ca_V_1.1 splice variants without changing their current amplitudes or kinetics. This demonstrates the exquisite sensitivity of the voltage-sensor function to changes in the specific amino acid side chains independent of their charge. Our results further indicate the cooperativity of VSDs I and IV in determining the voltage-sensitivity of Ca_V_1.1 channel gating.

## Introduction

The dihydropyridine (DHP) receptor functions as voltage-sensor in skeletal muscle excitation-contraction (EC) coupling and as a slowly activating calcium channel. It was the first member of the voltage-gated calcium channel family (Ca_V_) that has been biochemically isolated [1], the first to be cloned [2], and the first pseudo-tetrameric cation channel for which the protein structure has been solved using cryo-electron microscopy [3]. Therefore, Ca_V_1.1 can fittingly be considered as the prototypical voltage-gated calcium channel. Nevertheless, due to its dual function in EC-coupling and as calcium channel, Ca_V_1.1 has unique gating-properties that set it apart from the other members of the Ca_V_ family. Most importantly, its currents are comparatively small and their activation is slow and requires depolarization to considerably more positive potentials than that of other Ca_V_ channels [4,5]. Also, until recently Ca_V_1.1 was the only member of its family that could not be functionally expressed in non-muscle cells. Therefore, in the past the functional analysis of Ca_V_1.1 has primarily been the domain of scientists studying skeletal muscle function in native cells. With the recent discovery that co-expression of the scaffolding protein STAC3 allows functional expression of Ca_V_1.1 also in mammalian non-muscle cells and Xenopus oocyes [6,7], analysis of the Ca_V_1.1 channel isoform has been opened to a considerably wider research community.

**Figure 1.**
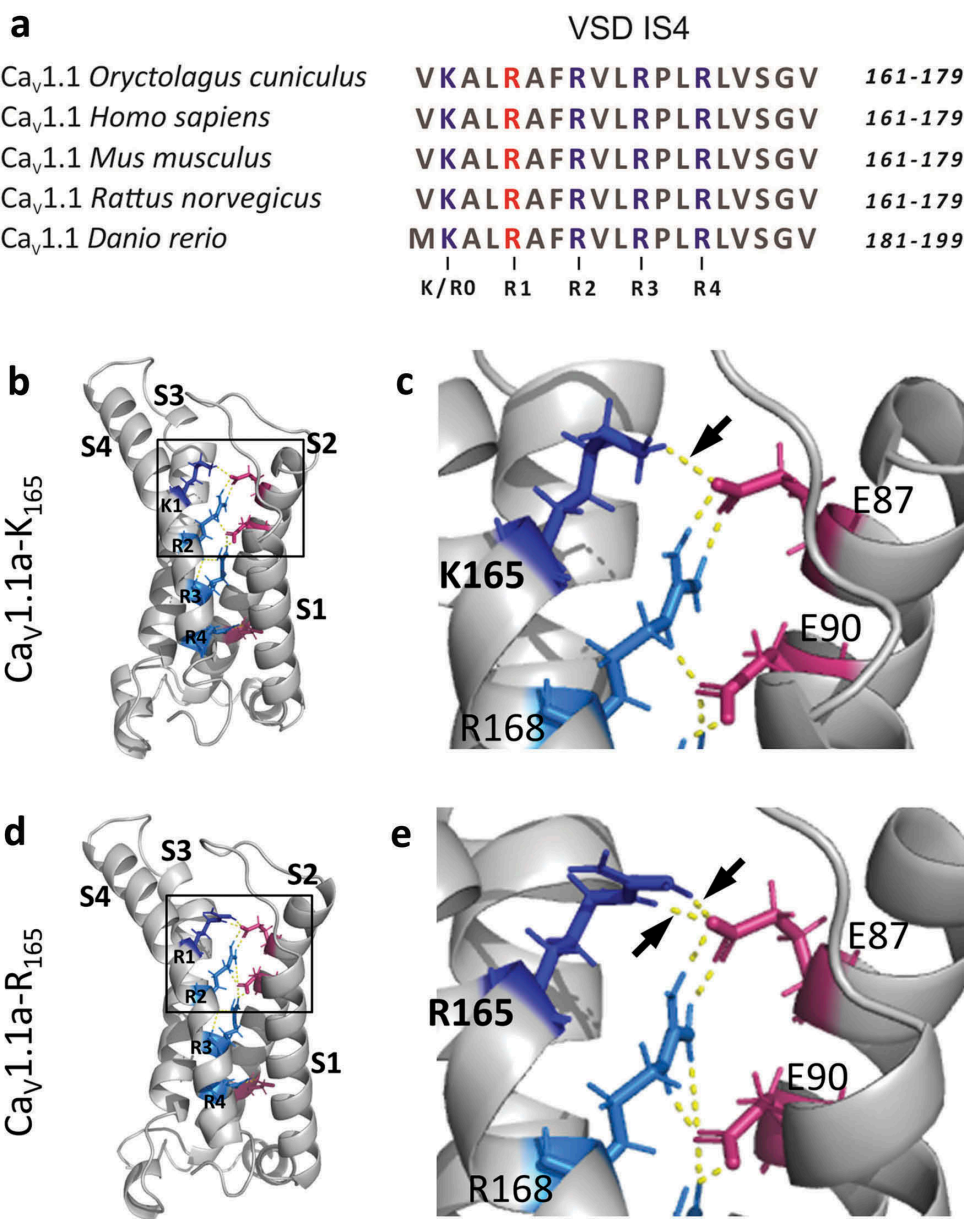
Structure of the first VSD of Ca_V_1.1. (a) Amino acid sequence alignment of the S4 transmembrane helices of the first VSD of Ca_V_1.1 from five vertebrate species; *Oryctolagus cuniculus* (UniProtKB, P07293), *Homo sapiens* (UniProtKB, Q13698), *Mus musculus* (UniProtKB, Q02789), *Rattus norvegicus* (UniProtKB, Q02485) and *Danio rerio* (UniProtKB, Q6RKB0). The conserved arginines in position R1 (R_165_ in the rabbit clone) are marked in red, the other four positively charged residues are marked in blue. (b–e) Structure models of the first VSDs of Ca_V_1.1a-K_165_ and Ca_V_1.1a-R_165_ in the up-state, based on the cryo-EM structure of Ca_V_1.1a [3,13]. (b, d) S1, S2, S3, and S4 denote the transmembrane helices; R/K1, R2, R3, and R4 the positively charged amino acids (blue) in every third position of S4 that transit the electric field across the membrane upon activation and deactivation. The frame indicates the area enlarged at right. (c, e) In the up-state the additional amino group of R165, compared to K165, enables the formation of an additional H-bond with the countercharge E87 in the S2 helix (arrows).

The original rabbit cDNA cloning revealed the primary amino acid sequence of Ca_V_1.1 and produced an expression plasmid (pCAC6) constructed from multiple cDNA fragments [2,8]. In a landmark study this plasmid was used to restore EC coupling and L-type calcium currents in dysgenic (Ca_V_1.1-null) myotubes [8], and subsequently it became the mother-construct for a multitude of derivatives used by scientists all around the world. However, as thoroughly documented in the original studies [2,8], the clones used to construct pCAC6 contained several variations, including one in the fourth transmembrane segment of the first voltage-sensing domain (IS4). In nucleotide position 494 a *G* (in clone pCCH810) or an *A* (in clone pCCH303) resulted in either an arginine (R) or a lysine (K), respectively, in amino-acid position 165. As clone pCCH303 had been used for construction of pCAC6 [8], the sequence encoded in this expression plasmid deviates from that of the published Ca_V_1.1 amino-acid sequence [2]. This cloning error affects the second of five positively charged residues in every third position of IS4, known to be important for the voltage-sensing process. As consensus numbering of voltage sensing charges in the first repeat of Ca_V_ channels starts with R/K0, amino acid 165 occupies the R1 position [9,10] (Figure 1). During depolarization and repolarization these positive gating charges move up and down through the plane of the membrane, thus effecting conformational changes in the voltage sensor domain (VSD) that cause activation and deactivation of the channel [9]. In the course of this process the positive S4 gating charges sequentially interact with negative counter-charges in the S2 helix of the VSDs (Figure 1(b)). Charge-neutralizing mutations of these highly conserved gating charges in various calcium channels lead to changes in voltage-sensitivity of activation [10–12]. The effects of charge-preserving mutations of these amino acids are less well known.

The classical skeletal muscle Ca_V_1.1a isoform is unique among the voltage-gated calcium channels in that membrane depolarization activates channel opening only at considerably more positive potentials than gating currents and depolarization-induced calcium release from the sarcoplasmic reticulum (i.e. EC coupling) [14,15]. This divergent voltage-dependence of EC-coupling and current activation depends on the developmentally regulated insertion of exon 29 and on a unique interaction of the outermost gating charges (R1 and R2) with countercharges in the fourth VSD [12,16–18]. In contrast, the characteristic slow activation kinetics of skeletal muscle calcium currents are determined by sequences in the first VSD [19,20]; thus suggesting that the specific properties of first and fourth VSDs of Ca_V_1.1 independently determine the kinetics and voltage-dependence of current activation, respectively [20]. However, the specific roles of individual gating charges in the first VSD are not known.

Because the physicochemical properties of the S4 gating charges are critical for determining the activation properties of voltage-gated calcium channels, and because the most commonly used Ca_V_1.1 expression plasmids all carry a conservative substitution of the second gating charge (R1) of the first VSD (R165K), we decided to correct this cloning error and analyze possible effects of the amino acid exchange on the current properties. Our results with the corrected Ca_V_1.1 construct show an approximately 12 mV right-shifted voltage-dependence of activation without affecting activation kinetics or current density. Thus, indicating that the first VSD contributes to the voltage-sensitivity of Ca_V_1.1 and that native channels are even less responsive to depolarization than previously shown with constructs carrying the R165K mutation.

## Results

**Figure 2.**
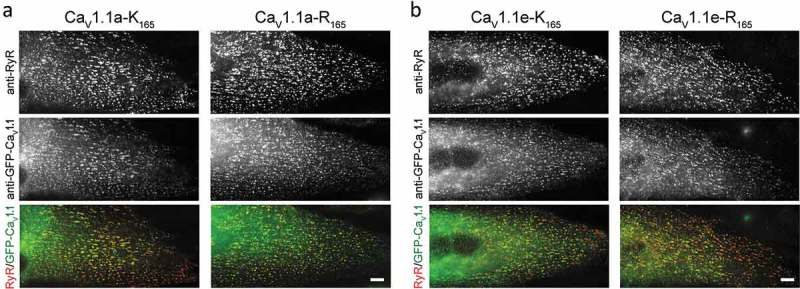
Expression and localization of Ca_V_1.1 constructs in dysgenic myotubes. (a) The conventional (K_165_) and the corrected (R_165_) constructs of the classical/adult Ca_V_1.1a splice variant including exon 29; (b) the conventional (K_165_) and the corrected (R_165_) constructs of the embryonic Ca_V_1.1e splice variant lacking exon 29. The GFP-tagged Ca_V_1.1 constructs were localized with anti-GFP (green) and co-stained with anti-RyR1 (red). Co-clustering of all Ca_V_1.1 constructs with RyR1 is indicative of their normal expression and targeting into the skeletal muscle triads. Scale bar, 10 µm.

The S4 transmembrane helices of the VSDs of voltage-gated calcium channels contain four to five positively charged amino acids (arginines or lysines, numbered R/K0, R1, R2, R3, R4) in every third position. Sequence comparison of the S4 transmembrane helix of the first VSD of high-voltage activated Ca_V_ channels shows the consensus sequence K0, R1, R2, R3, R4. Furthermore, in Ca_V_1.1 this consensus sequence is conserved across different species (Figure 1(a)). In contrast the widely used expression plasmid of the rabbit Ca_V_1.1 (pCAC6) and its numerous derivatives encode a lysine in position R1. We corrected this conservative substitution (K165R) in our GFP-tagged Ca_V_1.1 clone and compared the current properties of the original (Ca_V_1.1a-K_165_) and the corrected (Ca_V_1.1a-R_165_) channel variants in dysgenic (Ca_V_1.1-null) myotubes. Double immunofluorescence labeling with antibodies against GFP, to label the GFP-tagged Ca_V_1.1 constructs, and the type 1 ryanodine receptor (RyR1) confirmed that the corrected construct Ca_V_1.1a-R_165_ was normally expressed and incorporated into skeletal muscle triads (Figure 2(a)).

### Effects of the K165R substitution on the current parameters of the adult Ca_v_1.1a splice variant

**Figure 3.**
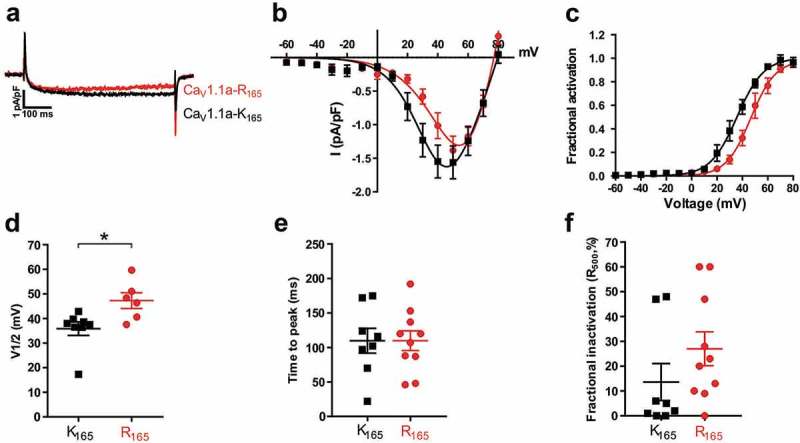
Correcting the R165K substitution in the VSD I of Ca_V_1.1a right-shifts the voltage-dependence of activation. (a) Representative calcium currents of Ca_V_1.1a-K_165_ (black) and Ca_V_1.1a-R_165_ (red) at the maximally activating test pulses. (b,c) I/V curves and the fractional activation plot show an 11.4 mV right shift of the voltage-dependence of activation of the corrected Ca_V_1.1a-R_165_ (red) compared to Ca_V_1.1a-K_165_ (black), but little difference in current density. (d) The scatter-plot of V½ shows a significant increase of the mean voltage at half-activation of Ca_V_1.1a-R_165_ (red) compared to Ca_V_1.1a-K_165_ (black) (mean±SE, N = 6–8, *P < 0.05, student *t*-test). (e,f) Scatter-plots of time to peak and fractional inactivation at the end of the 500-ms test pulse (R_500_) show that the K165R substitution did not affect the kinetics of the calcium currents.

First we analyzed the effects of the K165R substitution in the classical Ca_V_1.1a isoform, which is expressed in adult muscle and characterized by small calcium currents activated at comparably positive potentials [4,21]. Whole cell patch-clamp recordings of dysgenic myotubes transfected with either Ca_V_1.1a-K_165_ or Ca_V_1.1a-R_165_ revealed slowly-activating calcium currents of similar magnitude but distinct voltage-dependence of activation (Figure 3(a,b)). The current to voltage plots (I/V-curves) and the conductance to voltage plots (G/V-curves) for the corrected Ca_V_1.1a-R_165_ were shifted to more positive potentials, compared to those of the original Ca_V_1.1a-K_165_ construct (Figure 3(b,c)). The mean voltage at half-maximal activation for Ca_V_1.1a-K_165_ was 35.9 ± 2.8 mV (N = 8) and for Ca_V_1.1a-R_165_ 47.3 ± 2.5 mV (N = 6); the difference was statistically significant (P = 0.019) (Figure 3(d), Table 1). The mean values for time-to-peak, peak current density, and fractional inactivation at the end of the 500-ms test pulse (R_500_) did not significantly differ between the two constructs (Figure 3(b,e,f)). Thus, the charge-preserving substitution of R1 (K165R) in the first VSD of Ca_V_1.1a resulted in an 11.4 mV right-shift of the voltage-dependence of activation without affecting the kinetics or amplitude of the whole-cell calcium current.

**Table 1.** Properties of calcium currents from Ca_V_1.1a-K_165_ and Ca_V_1.1a-R_165._

Parameters	Ca_V_1.1a-K_165_	Ca_V_1.1a-R_165_	Significance (*p*-value)
*I*_peak_ (pA/pF)	−1.7 ± 0.3	−1.4 ± 0.2	0.41
*G*_max_ (nS/nF)	69.0 ± 10.5	96.9 ± 13.3	0.17
*V*_1/2_ (mV)	35.9 ± 2.8	47.3 ± 2.5	0.019 *
*k*_act_ (mV)	8.9 ± 0.9	8.6 ± 0.6	0.76
*V*_rev_ (mV)	79.7 ± 2.0	77.9 ± 0.9	0.37
Time to peak (ms)	109.8 ± 17.9	109.8 ± 14.3	0.998
*R*_500_ (%)	13.6 ± 7.4	27.1 ± 6.8	0.20
*n*(*n Ipeak*)	8(8)	6(10)	–
Parameters	Ca_V_1.1e-K_165_	Ca_V_1.1e-R_165_	Significance (*p*-value)
*I*_peak_ (pA/pF)	−9.1 ± 1.3	−9.3 ± 1.9	0.93
*G*_max_ (nS/nF)	160.6 ± 17.1	195.6 ± 38.2	0.42
*V*_1/2_ (mV)	6.9 ± 2.4	19.1 ± 1.3	0.0006 ***
*k*_act_ (mV)	5.2 ± 0.6	4.7 ± 0.4	0.49
*V*_rev_ (mV)	72.2 ± 1.9	83.9 ± 1.0	0.00013 ***
Time to peak (ms)	63.8 ± 17.1	69.9 ± 14.3	0.78
*R*_500_ (%)	38.1 ± 5.4	25.9 ± 4.5	0.11
*n*(*n Ipeak*)	8(8)	8(8)	–

All data are presented as mean ± SE. P-values were calculated using the student *t*-test. * p < 0.05, *** p < 0.001.

### Effects of the K165R substitution on the current parameters of the embryonic Ca_v_1.1e splice variant

**Figure 4.**
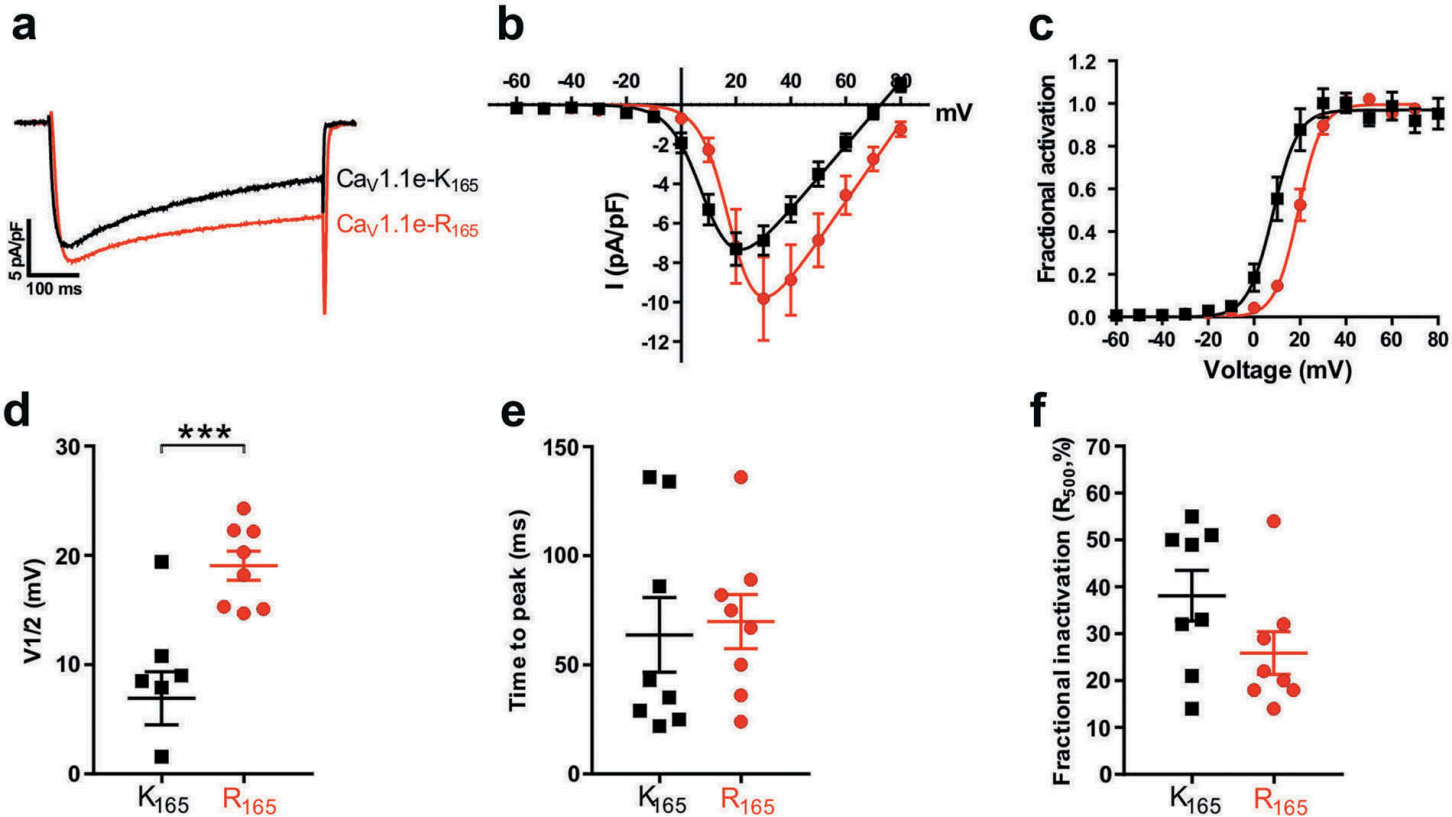
The correction of the R165K substitution in the VSD I of Ca_V_1.1e right-shifts the voltage-dependence of activation. (a) Representative calcium currents of Ca_V_1.1e-K_165_ (black) and Ca_V_1.1e-R_165_ (red) at the maximally activating test pulses. (b,c) I/V curves and the fractional activation plot show a >12 mV right shift of the voltage-dependence of activation of the corrected Ca_V_1.1e-R_165_ (red) compared to Ca_V_1.1e-K_165_ (black), with only an insignificant increase in current density. (D) The scatter-plot of the V½ shows a significant increase of the mean voltage at half-activation of Ca_V_1.1e-R_165_ (red) compared to Ca_V_1.1e-K_165_ (black) (mean±SE, N = 8, ***P < 0.001, student *t*-test). (e,f) Scatter-plots of time to peak and fractional inactivation at the end of the 500-ms test pulse (R_500_) show that the K165R substitution does not affect the kinetics of the calcium current.

Exclusion of exon 29 occurs in the embryonic Ca_V_1.1 splice variant [18,21]. The lack of the 19 amino acids encoded by exon 29 in the extracellular loop connecting S3 and S4 in the fourth VSD results in Ca_V_1.1e channels with substantially larger amplitudes and left-shifted voltage-dependence of current activation [12,16]. To examine whether, and if so how, this splice variant is affected by the K165R substitution, we generated the corresponding construct (Ca_V_1.1e-R_165_), expressed it in dysgenic myotubes and analyzed its whole-cell current properties. Again, normal expression and targeting into skeletal muscle triads was confirmed using double immunofluorescence labeling in dysgenic myotubes (Figure 2(b)). Consistent with published data, the original version of the embryonic Ca_V_1.1e-K_165_ variant gave rise to currents with >5-fold increased peak current densities and a >27 mV left-shifted voltage-dependence of activation, compared to the adult splice variant (Ca_V_1.1a-K_165_) reported above (compare Figures 3 and 4). Kinetics of activation and inactivation were faster in Ca_V_1.1e-K_165_ compared to Ca_V_1.1a-K_165_, as evident by a substantially reduced time-to-peak and a slightly increased fractional inactivation, respectively (Table 1). Although the overall current properties of the embryonic variants (Ca_V_1.1e-K_165_ and Ca_V_1.1e-R_165_; Figure 4) substantially differed from those of the adult variants (Ca_V_1.1a-K_165_ and Ca_V_1.1a-R_165_; Figure 3), the K165R substitution had similar specific effects on the voltage-dependence of activation in both the embryonic and the adult splice variants (Figure 4). The I/V- and the G/V-curves for the corrected Ca_V_1.1e-R_165_ were shifted by 12.2 mV to more positive potentials, relative to those of Ca_V_1.1e-K_165_ (Figure 4(b,c)). The mean voltage of half-maximal activation for the original Ca_V_1.1e-K_165_ was 6.9 ± 2.4 mV (N = 8) and for the corrected Ca_V_1.1e-R_165_ 19.1 ± 1.3 mV (N = 8); the difference was statistically highly significant (P < 0.001) (Table 1). Again, the mean values for time-to-peak, peak current density, and fractional inactivation at the end of the 500-ms test pulse (R_500_) were not significantly different between Ca_V_1.1e-K_165_ and Ca_V_1.1e-R_165_ (Table 1). Thus, irrespective of their distinct basal gating properties, the K165R substitution of R1 in the embryonic Ca_V_1.1e splice variant results in a similarly large and specific effect on its voltage-dependence of activation as in the adult/classic Ca_V_1.1a splice variant.

## Discussion

The primary question motivating this study was, whether the gating properties of the rabbit Ca_V_1.1 clone containing the R165K substitution, that we and most other labs are working with, actually represent those of the native skeletal muscle calcium channel. Already the early work in *dysgenic* myotubes reconstituted with the rabbit Ca_V_1.1 clone established the similarity of its biophysical properties with currents recorded in wildtype myotubes. Most importantly, like calcium currents in native skeletal muscle cells, *dysgenic* myotubes reconstituted with pCAC6 displayed the characteristic slow activation kinetics, right-shifted voltage-dependence of activation, and relatively small current amplitudes [8]. Therefore, the expectation was that, if the currents of the corrected Ca_V_1.1a-R_165_ differed at all from the original Ca_V_1.1a-K_165_, the differences would be rather small. Moreover, the exchange of an arginine with a lysine represents a conservative substitution maintaining the positive charge in the R1 position of the VSD. Nevertheless, due to the critical role of the positively charged S4 residues in voltage-dependent activation of the channel, also small changes affecting the movement of the gating charges through the electric field across the membrane can have a significant impact on gating properties. For example, previously we demonstrated that the charge-maintaining substitution of D1196E, a residue acting as counter-charge for R1 and R2 during activation of the fourth VSD, caused a >20 mV right-shift of the voltage-dependence of activation and a 5-fold reduction of current density [12]. Finally, knowing possible differences between the constructs based on pCAC6 and those with the correct IS4 sequence, gained heightened urgency, because of the renewed interest in studying Ca_V_1.1 in the wake of the discovery that STAC3 enables its functional expression in non-muscle cells [6,7,22].

Our results demonstrate that the substitution of K165 to R specifically caused a ~12 mV shift of the voltage-dependence of activation to more positive potentials. Thus, Ca_V_1.1 with the correct IS4 sequence requires even higher depolarizations for the activation of calcium currents than previously shown with the constructs derived from rabbit pCAC6. This right-shifted voltage-dependence further corroborates the notion that during physiological activation with trains of action potentials, Ca_V_1.1 currents will hardly activate and that in adult skeletal muscle calcium influx through Ca_V_1.1 is dispensable for normal muscle function [23,24]. Regarding the accuracy of previous work, however, the observed differences in voltage-dependence are negligible, because due to different recording conditions and experimental systems the range of reported values for the voltage-dependence of activation is considerably larger than the difference due to the amino acid substitution in the first VSD. Should from now on everybody be using the corrected versions of Ca_V_1.1? Not necessarily. As noted above, this is only one of many experimental factors affecting the recorded voltage-dependence of channel gating. Therefore, the absolute values are of lesser importance than relative differences between channel isoforms and mutants analyzed under the same experimental conditions. Thus, comparability with previous studies, by continuing working with the same expression constructs, may be of greater value than the fact that the absolute values are off by about 12 mV.

Nonetheless, the observed effect on the voltage-dependence of activation due to the K165R substitution is of considerable interest in its own right. First of all, it demonstrates that for the function of the gating charges in the voltage sensor not only the positive charge itself is important, but also the other physicochemical properties of the particular amino acid side chains. For example, the side chain of arginine has an additional amino group which can form additional H-bonds, and these differences probably affect the molecular interactions that facilitate the transition of the gating charges through the gating pore. Our data indicate that this is the case for the K165R substitution in the R1 position of the first VSD. The structure model of the first VSD of Ca_V_1.1a in the up-state indicates that R165 forms two H-bonds with E87 in the IS2 helix, whereas K165 forms only a single H-bond with E87 (Figure 1(b–e)). Similar differences in the interactions of K or R in position 165 with other partners within VSD I are likely to occur also in the resting states. Differences in one or more of these interactions may facilitate the voltage sensor transition of Ca_V_1.1a-K_165_ into the activated state and consequently cause the observed shift in the voltage-dependence of activation between Ca_V_1.1-K_165_ and Ca_V_1.1-R_165_ channels.

Secondly, the observed shift in voltage-dependence of activation resulting from the K165R substitution demonstrates that the first VSD of Ca_V_1.1 contributes to the voltage-sensitivity of channel gating. This was unexpected because previous findings by us and others indicated that the first VSD primarily determines the slow activation kinetics of Ca_V_1.1 without affecting its voltage-dependence [19,20]. Surprisingly, here we observe an effect on voltage-dependence without a significant change of activation kinetics. Apparently, even within a single VSD different structures are involved in determining different aspects of channel gating. One possible mechanism could be that certain transitions of the S4 helix along its path from the deep resting state to the activated state are limiting the speed of this process, while others define the principal energy barrier, which needs to be overcome to activate channel opening. Our results indicate that R1 (R165) is critically involved in the latter process.

Previously we demonstrated that the insertion of 19 amino acids in the IVS3-S4 linker of Ca_V_1.1 had a major effect on voltage-dependence of activation and current amplitude [12,16]. Together with the purely kinetic effects of modifications in the first VSD this suggested a dichotomic model according to which the first VSD determines activation kinetics, whereas the fourth VSD determines voltage-dependence of activation and current amplitude [20]. That the separation of gating properties to distinct VSDs is not absolute, was already apparent by the observation that the striking differences in voltage-sensitivity and amplitude between Ca_V_1.1a and Ca_V_1.1e were accompanied by minor differences of their kinetic properties (compare Figures 3 and 4) [16]. The present finding further qualifies the notion of a strict separation of gating properties to distinct VSDs. For the first time we observe effects on voltage-dependence of activation but not on current kinetics in response to structural changes in the first VSD of Ca_V_1.1.

Do the modifications in the first and fourth VSD that right-shift voltage-dependence of activation act on the same downstream mechanism or are they separable processes? To answer this question we analyzed the effects of the K165R substitution in both Ca_V_1.1a and Ca_V_1.1e. Insertion of 19 amino acids encoded by exon 29 into the IVS3-S4 linker of Ca_V_1.1a displaces R1 and R2 from their countercharge D1196, thus causing the ~30 mV right-shift of current activation [12]. Notably, this has no effect on the voltage-dependence of depolarization-induced calcium release, resulting in the characteristic separation of the voltage-dependence of current activation from that of EC-coupling in Ca_V_1.1a, but not in Ca_V_1.1e [16]. Interestingly, here the K165R substitution right-shifted the voltage-dependence of both splice variants, and in Ca_V_1.1a the effects caused by insertion of exon 29 and by the K165R substitution were additive. Therefore, we conclude that the two effects are not mediated by a common molecular mechanism. The existence of distinct underlying mechanisms is further substantiated by our data showing that the insertion of exon 29 into the fourth VSD causes the shifted V_1/2_ plus a >5-fold decrease in I_peak_, whereas the K165R substitution in the first VSD causes the shift in V_1/2_ of activation without affecting current density.

Different contributions of two distinct VSDs to the voltage-dependence of activation supports an allosteric gating model, as opposed to an obligatory model, as has been proposed based on a voltage-clamp fluorometry study of Ca_V_1.2 in *Xenopus* oocytes [25]. If activation of the fourth VSD would be obligatory for channel gating, then the substantial right-shift caused by insertion of exon 29 in the fourth VSD of Ca_V_1.1a would mask the comparatively smaller effect caused by the K165R substitution in the first VSD. In that case, K165R would be expected to show a right-shift in Ca_V_1.1e, but not in Ca_V_1.1a. On the other hand, if multiple VSDs each contribute different amounts of energy to gating, as proposed in the allosteric gating model, the impact on voltage-dependence of activation of simultaneous modifications in different participating VSDs would be additive, as we observed with the combined K165R substitution in the first VSD and inclusion of exon 29 in the fourth VSD.

In conclusion, correcting a conserved amino acid substitution in the first VSD of two functionally distinct splice-variants of the rabbit Ca_V_1.1 plasmid revealed an ~12 mV right-shift in the voltage-dependence of current activation that is of limited consequence for the interpretation of previous work using constructs with the incorrect sequence, but elucidates the role of the first VSD, and its functional cooperation with the fourth VSD, in determining the gating properties of Ca_V_1.1 calcium channels.

## Materials and methods

### Expression plasmids

Cloning procedures for GFP-Ca_V_1.1a-K_165_ and GFP-Ca_V_1.1e-K_165_ were previously described [16,26]. In order to generate GFP-Ca_V_1.1a-R_165_, K165 was corrected by SOE-PCR. Briefly, nt 1–1113 of Ca_V_1.1 were PCR amplified with overlapping primers introducing the point mutation A > G at position nt 494 in separate PCR reactions using GFP-Ca_V_1.1a-K_165_ as template. The two separate PCR products were then used as templates for a final PCR reaction with flanking primers to connect the nucleotide sequences. This fragment was then SalI/EcoRI digested and cloned into the respective sites of GFP-Ca_V_1.1a-K_165_, yielding GFP-Ca_V_1.1a-R_165_. To generate GFP-Ca_V_1.1e-R_165_, nt 2654-4488 of the Ca_V_1.1e coding sequence were isolated from GFP-Ca_V_1.1e-K_165_ by digestion with XhoI and BglII and inserted in the corresponding sites of GFP-Ca_V_1.1a-R_165_, yielding GFP-Ca_V_1.1e-R_165_. Sequence integrity of the newly generated constructs was confirmed by sequencing (MWG Biotech, Martinsried, Germany).

### Cell culture and transfections

Myoblasts of the dysgenic (mdg/mdg) cell line GLT were transfected 4 days after plating using FuGENE-HD transfection reagent (Promega). Transfected myotubes, identified by GFP fluorescence, were analyzed 3 or 4 days after transfection. The auxiliary calcium channel subunits α2δ-1, β1a, and γ_1_, along with the STAC3 protein and ryanodine receptor, are endogenously expressed by GLT myotubes, enabling functional membrane incorporation of the channel constructs in the triad junction.

### Immunofluorescence and antibodies

Paraformaldehyde-fixed cultures were immunolabeled as previously described [17,27] with rabbit polyclonal anti-GFP (1:10,000; Molecular Probes) and mouse monoclonal anti-RyR (34-C; 1:1000; Alexis Biochemicals) and fluorescently labeled with goat anti-rabbit Alexa-488 and secondary goat anti-mouse Alexa-594 (1:4000), respectively. Thus, the anti-GFP label and the intrinsic GFP signal were both recorded in the green channel. Samples were observed using a 63X, 1.4 NA objective Axioimager microscope (Carl Zeiss Inc.) and 14-bit images were captured with a cooled charge-coupled device camera (SPOT; Diagnostic Instruments) and Metaview image-processing software (Universal Imaging). Image composites were arranged in Adobe Photoshop CS6 (Adobe Systems INC.) and linear adjustments were performed to correct black level and contrast.

### Electrophysiology and data analysis

Calcium currents were recorded with the whole-cell patch-clamp technique in voltage-clamp mode using an Axopatch 200A amplifier (Axon Instruments). Patch pipettes (borosilicate glass; Science Products) had resistances between 1.8 and 3 MΩ when filled with (mM) 145 Cs-aspartate, 2 MgCl2, 10 HEPES, 0.1 Cs-EGTA, and 2 Mg-ATP (pH 7.4 with CsOH). The extracellular bath solution contained (mM) 10 CaCl2, 145 tetraethylammonium chloride, and 10 HEPES (pH 7.4 with tetra-ethylammonium hydroxide). Data acquisition and command potentials were controlled by pCLAMP software (Clampex version 10.2; Axon Instruments); analysis was performed using Clampfit 10.7 (Axon Instruments) and SigmaPlot 12.0 (SPSS Science) software. The current-voltage dependence was fitted according to

$$I=\;\;G_{max}*(V-V_{rev})/(1+exp(-(V-V_{1/2})/\;k))$$

where G_max_ is the maximum conductance of the L-type calcium currents, V_rev_ is the extrapolated reversal potential of the calcium current, V_1/2_ is the potential for half maximal conductance, and *k* is the slope. The conductance was calculated using G = (− I * 1000)/(V_rev_ − V), and its voltage dependence was fitted according to a Boltzmann distribution:

$$G=\;\;G_{max}/\left(1+exp\left(-\left(V-V_{1/2}\right)/\;k\right)\right)$$

### Statistical analysis

All four experimental groups were analyzed in transiently transfected cells from 4 to 5 independent cell passages. The R165 and K165 variants of Ca_V_1.1a and Ca_V_1.1e were recorded in parallel in cells of the same passage. The means, standard errors (SE), and p-values were calculated using the student *t*-test, 2-tailed, with significance criteria p < 0.05 *, p < 0.01 **, and p < 0.001 *** (see Table 1).
